# Fibroblast growth factor receptor signaling in pediatric B-cell precursor acute lymphoblastic leukemia

**DOI:** 10.1038/s41598-018-38169-z

**Published:** 2019-02-12

**Authors:** Isabel S. Jerchel, Alex Q. Hoogkamer, Ingrid M. Ariës, Judith M. Boer, Nicolle J. M. Besselink, Marco J. Koudijs, Rob Pieters, Monique L. den Boer

**Affiliations:** 1 Department of Pediatric Oncology, Erasmus Medical Center – Sophia Children’s Hospital, Rotterdam, The Netherlands; 2 Center for Personalized Cancer Treatment, University Medical Center Utrecht, Utrecht, The Netherlands; 3 Center for Molecular Medicine, Cancer Genomics Netherlands, Division Biomedical Genetics, University Medical Center Utrecht, Utrecht, The Netherlands; 4 Dutch Childhood Oncology Group (DCOG), The Hague, The Netherlands; 5 Princess Máxima Center for Pediatric Oncology, Utrecht, The Netherlands; 6 Present Address: Princess Máxima Center for Pediatric Oncology, Heidelberglaan 25, 3584 CS Utrecht, The Netherlands

## Abstract

The FGF receptor signaling pathway is recurrently involved in the leukemogenic processes. Oncogenic fusions of FGFR1 with various fusion partners were described in myeloid proliferative neoplasms, and overexpression and mutations of FGFR3 are common in multiple myeloma. In addition, fibroblast growth factors are abundant in the bone marrow, and they were shown to enhance the survival of acute myeloid leukemia cells. Here we investigate the effect of FGFR stimulation on pediatric BCP-ALL cells *in vitro*, and search for mutations with deep targeted next-generation sequencing of mutational hotspots in *FGFR1, FGFR2*, and *FGFR3*. In 481 primary BCP-ALL cases, 28 samples from 19 unique relapsed BCP-ALL cases, and twelve BCP-ALL cell lines we found that mutations are rare (4/481 = 0.8%, 0/28 and 0/12) and do not affect codons which are frequently mutated in other malignancies. However, recombinant ligand FGF2 reduced the response to prednisolone in several BCP-ALL cell lines *in vitro*. We therefore conclude that FGFR signaling can contribute to prednisolone resistance in BCP-ALL cells, but that activating mutations in this receptor tyrosine kinase family are very rare.

## Introduction

Despite generally excellent outcome for children with pediatric B-cell precursor acute lymphoblastic leukemia (BCP-ALL), therapeutic options are limited for refractory or relapsed cases. Small molecule inhibitors of oncogenic pathways, such as those targeting ABL-, MEK, and JAK kinase families, have shown (pre-)clinical efficacy and may be added to chemotherapy in the future. Here we studied the role of fibroblast growth factor receptor (FGFR) signaling in pediatric BCP-ALL.

FGFs are not only essential in hematopoietic development, but also influence bone formation by regulating expansion and stemness of mesenchymal stromal cells^1,2^. FGF2 is expressed in the hematopoietic and stromal compartments of the bone marrow^1^. FGFR fusions have been described in atypical myeloproliferative disorders which may involve lymphoma or present as BCP-ALL^3^. High *FGFR3* expression is a hallmark of t(4;14)-positive multiple myeloma, and activating mutations are recurrently detected in this context^4–6^. Furthermore, FGFR signaling has been shown to contribute to drug resistance in chronic myeloid leukemia^7^. Thus, oncogenic FGFR signaling contributes to hematologic malignancies, but so far information on its role in pediatric BCP-ALL is limited. We therefore applied a customized targeted sequencing approach for mutational hotspots of *FGFR1, FGFR2*, and *FGFR3* and evaluated the effect of recombinant FGF2 on *in vitro* prednisolone sensitivity in BCP-ALL cell lines.

## Methods

### Patient material and cell lines

The study comprised children with BCP-ALL with age at diagnosis ranging from 0 to 18 years. Use of excess materials for research purposes has been approved by the Institutional Review Board of the University Medical Center Rotterdam on August 2nd, 2004, IRB approval file number MEC 2004-203. Written informed consent was obtained from parents or legal guardians and studies were conducted in accordance with the Declaration of Helsinki. Clinical characteristics were kindly provided by the Dutch Childhood Oncology Group (the Hague, Netherlands). Mononuclear cells were isolated using density gradient centrifugation with Lymphoprep (Axis Shield, Norway) as previously described^8^. All samples contained at least 90% leukemic blasts. Cytogenetic subtypes were determined by karyotype, fluorescence *in-situ* hybridization (FISH), and/or fusion-gene specific PCR. *BCR-ABL1*-like cases were identified using microarray gene expression profiling by the means of a 110 probeset classifier^9^. Leukemic cell lines (697, HAL01, Kasumi-2, LAZ-221, MHH-CALL2, MHH-CALL3, MHH-CALL4, Nalm-6, RCH-ACV, Reh, Sup-B15, Tom-1) were obtained from the German Collection of Microorganisms and Cell Cultures (DSMZ, Germany) and routinely validated by DNA fingerprinting. Unless indicated otherwise, all cell lines were cultured in RPMI-1640 supplemented with 10 to 20% fetal calf serum and penicillin, streptomycin and fungizone (all Life technologies).

### *In vitro* cytotoxicity assays

The IC_50_ of prednisolone was determined in an MTS assay for cell lines and an MTT assay for primary ALL cells based on a six-step range from 250 to 0.008 µg/mL (Nalm6, 697, RCH-ACV, MHH-CALL3) or 1 to 0.003 µg/mL (MHH-CALL2, MHH-CALL4, SupB15, Tom1). For these assays, cells were tested for viability in the prednisolone concentration range with or without 50 ng/mL recombinant human FGF2 (Bio-Rad) and with or without 1 µM AZD4547 (Selleckchem). After four days incubation at 37 °C and 5% CO_2_, cell viability was evaluated using MTS/MTT. IC_50_-values were calculated as the prednisolone concentration at which 50% of cells survived relative to the respective prednisolone-free control of each condition. To avoid redundancies with abundant cytokines in fetal calf serum (FCS), these assays were performed under reduced serum conditions (2% FCS for cells usually cultured in 10% FCS, and 4% FCS for those usually cultured with 20% FCS). Sensitivity towards AZD4547 was measured similarly, but at normal serum concentrations (10 µM–0.3 nM).

### Sequencing and code availability

**Figure 1:**
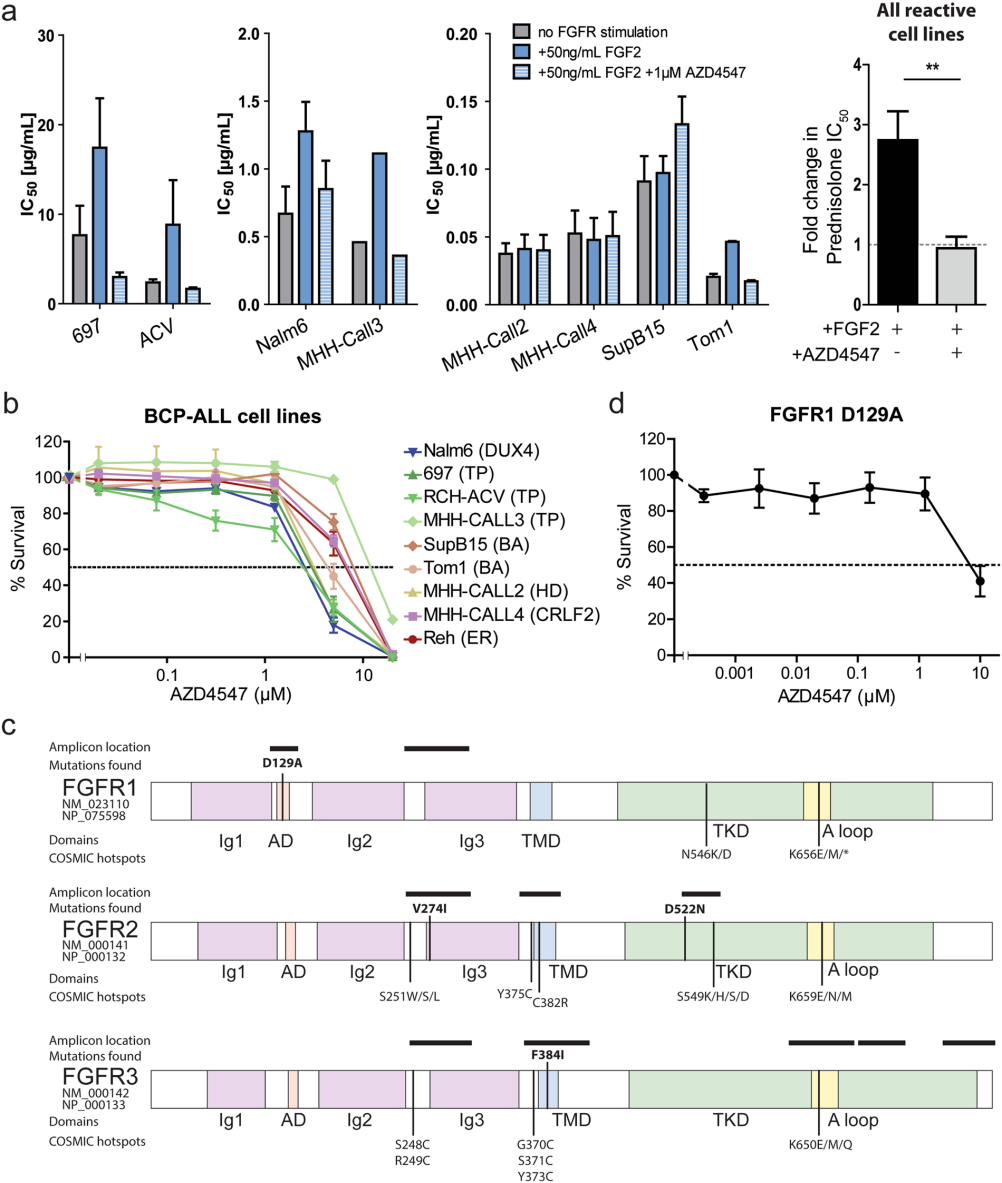
FGFR signaling is not essential in pediatric BCP-ALL cells, but can reduce prednisolone sensitivity. (**a**) Effect of 50 ng/mL recombinant human FGF2 on *ex vivo* response to prednisolone in BCP-ALL cell lines and reversal by the FGFR-inhibitor AZD4547 (1 µM). Left panels: prednisolone IC_50_-concentrations are depicted for each cell line. IC_50_-values for the combination of FGF2 and AZD4547 were calculated after correction for cytotoxic effects of FGF2 and AZD4547 as single agents on leukemic cell survival (see also panel (b); corresponding dose-response curves are shown in Supplemental Fig. 1b). Bars represent the mean ± SEM of Nalm6 (n = 3), 697 (n = 3), RCH-ACV (n = 3), SupB15 (n = 3), Tom1 (n = 2), MHH-CALL2 (n = 4), MHH-CALL3 (n = 1), and MHH-CALL4 (n = 4). Right panel: mean fold change of prednisolone IC_50_-values in FGF2-responsive cell lines (Nalm6, 697, RCH-ACV, Tom1, and MHH-CALL3), **p = 0.0012 by Wilcoxon matched pairs test. (**b**) *Ex vivo* response of BCP-ALL cell lines to the FGFR-inhibitor AZD4547. Dashed line: 50% survival; Cytogenetic subtypes are indicated in brackets: DUX4: *DUX4-*rearranged leukemia, TP: *TCF3-PBX1*-rearranged, BA: *BCR-ABL1-*rearranged, HD: high hyperdiploid karyotype, CRLF2: high *CRLF2* expression, ER: *ETV6-RUNX1*-rearranged. (**c**) Schematic representation of protein domains of the *FGFR* genes and the observed variants. Black bars indicate the regions covered by targeted amplicon sequencing. Variants printed in bold above the scheme represent variants observed in this study, variants below represent those frequently reported in the COSMIC database. Ig: Immunoglobulin-like domain, AD: acidic domain, TMD: transmembrane domain, TKD: tyrosine-kinase domain, A-loop: activation loop. (**d**) *Ex vivo* response of primary BCP-ALL cells carrying an FGFR1 D129A mutation.

DNA was isolated using Trizol reagent (Life Technologies), or in case of three cell lines using the DNeasy Kit (Qiagen). For TruSeq Custom Amplicon sequencing (Illumina, USA), sequencing libraries were prepared from 100–250 ng genomic DNA. Successful library preparation was confirmed using the Labchip GX genomic analyzer (Caliper Life Sciences Benelux N.V., the Netherlands). Samples were then pooled equimolarly and sequenced on an Illumina MiSeq in paired-end reads of 250 bp each. Targeted regions and the analysis script can be provided upon request. Next, sequence reads were aligned to the 1000 genomes human reference sequences (version b37, GATK resource bundle, Broad Institute, USA) using BWA v0.7.10^10^ and GATK indel realigner v.3.3-0. Single nucleotide variants were called with Freebayes v0.9.18–24^11^, Varscan v.2.3.7^12^, Bcftools v1.0^13^, and GATK v3.3-0^14^. The resulting variant call format files were annotated using snpEff and snpSift v.4.1a^15^ and dbNSFP v.2.7^16^. For reliable detection of high-confidence mutations, variants were filtered based on several criteria: For each sample, variants were excluded if they were reported by only one caller, coverage was <100 reads, or <20 reads supported the variant allele. Also variants likely to result from amplification or sequencing errors were excluded, i.e. those that occur repeatedly (n ≥10) but in a low fraction of reads within all samples (variant allele frequency (VAF) within each sample <2%) and those with an unequal distribution between runs. Furthermore, variants were only considered if they were non-synonymous, unlikely to be germline variants and not known SNPs. Mutational hotspots are indicated in Fig. 1c and were obtained from literature and the COSMIC database (v79, released 14^th^ November 2016)^6^. All data and analysis code can be made available upon request.

## Results

### FGF2 stimulation reduces prednisolone sensitivity in a subset of BCP-ALL cells

FGF2-mediated signaling through FGFR3 has been implicated in therapy resistance in AML^7^. We therefore evaluated the resistance-inducing effect of FGF2 on prednisolone cytotoxicity, a core component of ALL chemotherapy, using *in vitro* drug sensitivity assays. To avoid redundancies with abundant cytokines in fetal calf serum (FCS), these assays were performed under reduced serum conditions (2% FCS for cells usually cultured in 10% FCS, and 4% FCS for cells usually cultured with 20% FCS). The IC_50_ of prednisolone was determined in an MTS assay for eight BCP-ALL cell lines see also Supplemental Fig. 1). In five of the eight tested cell lines exposure to 50 ng/mL recombinant human FGF2 (Bio-Rad, USA) resulted in on average two- to three-fold higher IC_50_-values for prednisolone (Nalm6, 697, RCH-ACV, Tom1, and MHH-CALL3, Fig. 1a). The resistance induced by the ligand FGF2 was reversed by exposure to 1 µM of FGFR inhibitor AZD4547. AZD4547 as single agent reduced viability in BCP-ALL cell lines only at higher concentrations (5 to 20 µM, Fig. 1b), except for Nalm6 and RCH-ACV cell line (20–40% cell death induced at 1 µM AZD4547). The prednisolone IC_50_-values were corrected for the cytotoxic effect of AZD4547 as single agent and therefore reflect the synergistic effect of this FGFR inhibitor on the response to prednisolone.

### Absence of know oncogenic mutations in the genes FGFR1, -2, and -3

Using the TruSeq Custom Amplicon technique as previously described (ref.^17^ and Supplementary information), we screened for mutations in 481 primary BCP-ALL cases at initial diagnosis, in 28 BCP-ALL relapse samples (representing 19 unique patients) and in twelve BCP-ALL cell lines. The regions covered by the custom amplicons are indicated in Fig. 1c, and were selected to cover regions of functional relevance (e.g. the tyrosine kinase domain and transmembrane domain) and previously reported mutational hotspots of *FGFR1, FGFR2*, and *FGFR3* (Fig. 1c, hotspots are indicated below each gene). In 481 unique patients we found 4 non-synonymous, likely somatic FGFR variants. These variants were detected in samples taken at initial diagnosis, one FGFR1 (D129A) mutation, two FGFR2 mutations (V274I and D522N), and one FGFR3 (F384I) mutation (Fig. 1c, above gene schematics). No mutations were detected in the B-cell precursor ALL cell line panel or in the 28 relapse samples. The FGFR1 D129A and FGFR2 V274I mutations were predicted to be damaging to the protein function by the SIFT, Polyphen, LRT, and MutationTaster tools^18^. However, it is important to note that these prediction tools were trained to detect deleterious mutations, and activating mutations could be predicted as benign. Variant allele frequencies (VAF) varied, with 8.4% for the FGFR2 D522N variant, 28.5% for the FGFR1 D129A variant, 40.8% for the FGFR2 V274I variant, and 52.9% for the FGFR3 F384I variant. None of these mutations represents a frequently observed pathogenic mutation, such as those reported in multiple myeloma, according to literature and the COSMIC database (v79 released 14-Nov-16, indicated below each gene in Fig. 1c)^19^. Their phenotypic effect is unclear, however, all mutations locate within functional domains of the FGFR proteins (colored boxes).

FGFR variants occurred in two *ETV6-RUNX1*-rearranged cases, one *BCR-ABL1*-like with a *JAK2* translocation, and one high hyperdiploid, and high white blood cell count (≥50 cells/nL) was found in two out of four cases, high minimal residual disease (percentage blasts in bone marrow on day 33 of therapy ≥0.1%) in one out of three cases (Supplemental Table 1 one case without data available). The *JAK2*-translocated case harboring an FGFR1 D129A mutation experienced a relapse 1 year after diagnosis. In conclusion, FGFR variants occurred in various subtypes and patients did not consistently present with poor or good prognostic factors. Notably, the FGFR inhibitor was not effective in primary leukemic cells carrying the D129A point mutation in FGFR1 (Fig. 1d). Unfortunately no viable leukemic cells were available for other mutated BCP-ALL cases.

## Discussion

Here we screened 481 pediatric BCP-ALL cases at initial diagnosis, 19 relapse BCP-ALL cases, and 12 BCP-ALL cell lines for variants in *FGFR1, FGFR2*, and *FGFR3*. We found a low frequency of variants among patients at initial diagnosis, and no variants in the relapse cohort or in cell lines. These data indicate that FGFR mutations are very rare in pediatric BCP-ALL. Due to the low incidence and a small relapse cohort, it remains to be determined whether these rare mutations have an effect on the clinical prognosis. With the limited data available, we conclude that this does not seem to be the case.

The observed mutations are located within functional domains of the FGFR proteins: The FGFR1 D129A mutation affects the so-called acid box of the receptor, which has been shown to be essential for auto-inhibition and interaction with adhesion molecules^20,21^. The FGFR2 mutations V274I and D522N locate in the ligand-binding immunoglobulin-like (Ig) domain and the tyrosine kinase domain (TKD), respectively. They might therefore alter ligand-binding and kinase activation. Last, the FGFR3 F384I mutation is located in the transmembrane domain (TMD), which is frequently affected by mutations that cause aberrant receptor dimerization (e.g. by insertion of a cysteine). Of note, a rare F384L SNP at the same location has been reported and was also found in our screen. No functional consequences have been reported for this SNP, and the high similarity between leucine and isoleucine suggests no effect of the F384I variant on FGFR3 function^22^.

Given the previous implications of FGFR signaling in resistance, we evaluated its effect on the efficacy of prednisolone *in vitro*^7^. Our data indicate that the ligand FGF2 induced resistance to glucocorticoids which could be reversed by inhibition of FGFR. Traer *et al*. suggested that reactivation of the MAPK pathway by FGFR3 accounts for FGF-mediated resistance of *BCR-ABL1*-rearranged chronic myeloid leukemia cells to imatinib. In line with this observation, we and others have previously shown that MAPK pathway mediated signaling can also increase resistance towards prednisolone^23–25^. Downstream-located MAPK signaling may therefore be the mediator of FGF-induced glucocorticoid resistance, and could serve as therapeutic target for sensitization towards prednisolone in resistant cases.

Taken together, our data demonstrates that FGFR-activating lesions are rare in newly diagnosed pediatric BCP-ALL, and were not enriched at the time of relapse. Activating mutations which are frequently observed in multiple myeloma or solid cancers were not observed in 481 newly diagnosed nor in 19 relapsed BCP-ALL cases^19^. As FGFR activating mutations were virtually absent, FGF2 ligand-mediated activation of FGFR-signaling may be more relevant for the sensitivity of leukemic cells to prednisolone.

## Supplementary information

Supplementary Information

## Data Availability

Data can be made available upon request.
