# Supplementary material for: Fibroblast growth factor receptor signaling in pediatric B-cell precursor acute lymphoblastic leukemia
Source: Sci Rep. 2019 Feb 12;9:1875. doi: 10.1038/s41598-018-38169-z (PMC6372586; doi:10.1038/s41598-018-38169-z)

Supplementary Information for

Fibroblast growth factor receptor signaling in pediatric B-cell precursor acute lymphoblastic leukemia

Isabel S. Jerchel[1], Alex Q. Hoogkamer[1], Ingrid M. Ariës[1], Judith M. Boer[1], Nicolle J.M. Besselink[2,3], Marco J. Koudijs[2,3], Rob Pieters[4,5], and Monique L. den Boer[1,4,*]

[1] Department of Pediatric Oncology, Erasmus Medical Center – Sophia Children's Hospital, Rotterdam, the Netherlands; [2] Center for Personalized Cancer Treatment, University Medical Center Utrecht, the Netherlands; [3] Center for Molecular Medicine, Cancer Genomics Netherlands, Division Biomedical Genetics, University Medical Center Utrecht, the Netherlands; [4] Dutch Childhood Oncology Group (DCOG), The Hague, the Netherlands; [5] Princess Máxima Center for Pediatric Oncology, Utrecht, the Netherlands.

* Corresponding author: Prof. dr. M.L. den Boer
Current address:
Princess Máxima Center for Pediatric Oncology, Heidelberglaan 25, 3584 CS Utrecht
e-mail: m.l.denboer@prinsesmaximacentrum.nl
phone: +31 88 97 25064

Supplementary Tables

Supplementary Table 1 Characteristics of FGFR mutated cases.

Cytogenetic subtype	Mutated FGFR gene	Exact variant (GRCh37)	a. a. change	Coverage	Variant reads	VAF (%)	dbNSFP SIFT prediction	Age at diagnosis	WBC (/nL)	MRD d33	Event	EFS (years)	PRED *in vitro* IC_{50}
BAL	FGFR1	8:38285926 T/G	D129A	441	126	28.5	D	5	204	<0.1%	relapse	1.08	0.06μg/mL
ER	FGFR2	10:123258117 C/T	D522N	1494	125	8.4	T	3	13	0	-	8.32	0.35μg/mL
HD	FGFR2	10:123279612 C/T	V274I	864	352	40.8	D	5	10.5	>0.1%	-	6.35	N/A
ER	FGFR3	4:1806131 T/A	F384I	136	72	52.9	T	3	254.3	NA	-	8.49	0.04μg/mL

a.a.: amino acid change. VAF: variant allele frequency. WBC: white blood cell count. MRD d33: minimal residual disease after induction therapy including high dose glucocorticoid treatment; high MRD is defined as blast percentage in bone marrow >0.1%. EFS: follow-up time for event-free survival estimation. BAL: BCR-ABL1-like, ER: ETV6-RUNX1-positive, HD: High Hyperdiploid. dbNSFP SIFT abbreviations: D: damaging T: tolerated. PRED: prednisolone, N/A: Not available.

Supplementary Figures

Supplementary Figure 1: Dose-response curves corresponding to data presented in Fig. 1. **(a)** Cell survival compared to completely untreated cells, **(b)** cell survival relative to the respective stimulated or non-stimulated controls for each condition.

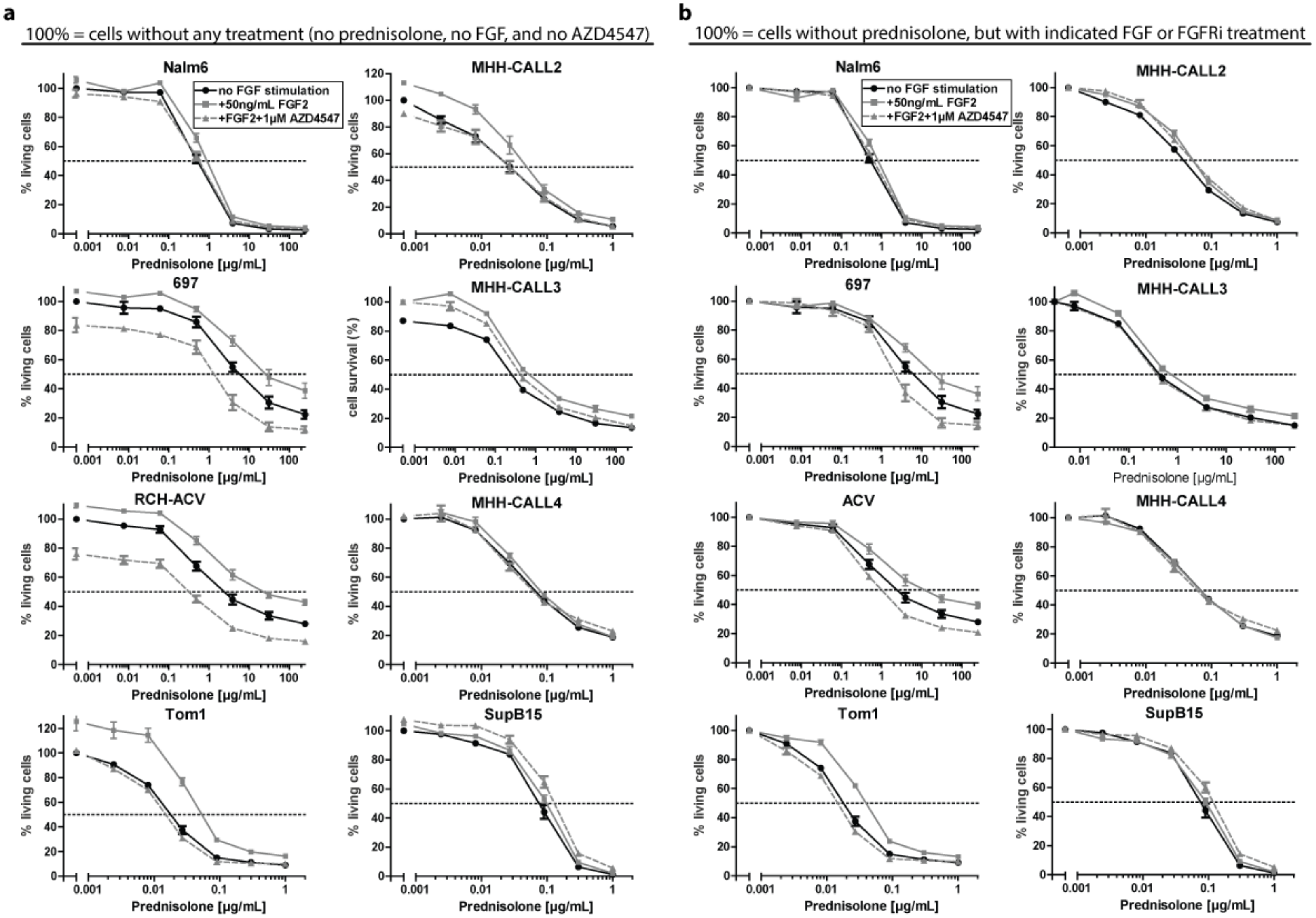

Supplement: Supplementary file 1 — Supplementary Information [file 41598_2018_38169_MOESM1_ESM.pdf]
